# Ag Nanoparticles Deposited onto BaTiO_3_ Aerogel for Highly Efficient Photodegradation

**DOI:** 10.3390/gels10060378

**Published:** 2024-05-31

**Authors:** Jun Wu, Wen Yan, Mengyuan Xie, Kai Zhong, Sheng Cui, Xiaodong Shen

**Affiliations:** 1 College of Materials Science and Engineering, Nanjing Tech University, Nanjing 211816, China; wujun890329@163.com (J.W.); 6395@njtech.edu.cn (W.Y.); 2 Jiangsu Collaborative Innovation Center for Advanced Inorganic Function Composites, Nanjing Tech University, Nanjing 211816, China; 3 CTC Nanjing Guocai Testing Limited Corporation, Nanjing 210046, China; xmy18862124378@163.com; 4 The 55th Research Institute of China Electronics Technology Group Corporation, Nanjing 211111, China; zhongkaicetc55@163.com

**Keywords:** BaTiO_3_ aerogel, 3D porous structural, noble metal deposition, photodegradation, methyl orange removal

## Abstract

Given the increasingly severe environmental problems caused by water pollution, the degradation of organic dyes can be effectively achieved through the utilization of photocatalysis. In this work, metal alkoxides and a combination of alcohol/hydrophobic solvents are employed to prepare BaTiO_3_ aerogels via a liquid-phase and template-free synthetic route. The preparation process of the aerogels solely entails facile agitation and supercritical drying, eliminating the need for additional heat treatment. The binary solvent of ethanol and toluene is identified as the optimal choice, resulting in a significantly enhanced surface area (up to 223 m^2^/g) and an abundant pore structure of BaTiO_3_ aerogels compared to that of the BaTiO_3_ nanoparticles. Thus, the removal efficiency of the BaTiO_3_ aerogel sample for MO is nearly twice as high as that of the BaTiO_3_ nanoparticles sample. Noble metal Ag nanoparticles’ deposition onto the BaTiO_3_ aerogel surface is further achieved via the photochemical deposition method, which enhances the capture of photogenerated electrons, thereby ensuring an elevated level of photocatalytic efficiency. As a result, Ag nanoparticles deposited on BaTiO_3_ aerogel can degrade MO completely after 40 min of illumination, while the corresponding aerogel before modification can only remove 80% of MO after 60 min. The present work not only complements the preparatory investigation of intricate aerogels but also offers a fresh perspective for the development of diverse perovskite aerogels with broad applications.

## 1. Introduction

Rapid and widespread industrialization worldwide is the primary cause of liquid waste generation by various industries. The textile industries contribute significantly to the global wastewater volume due to their enormous water consumption, thereby posing significant risks of water and soil pollution [1]. Textile industries consume a wide range of manufactured colorants and discharge a significant quantity of colored organic dyes, which have a negative impact on the photosynthetic function of plants and aquatic life [2,3,4]. The dyes utilized in the textile industry are stable under various environmental conditions, including exposure to light and fluctuations in temperature [3,5]. The persistence and resistance to degradation of dyes present significant challenges for wastewater treatment plants. Therefore, the photocatalytic approach for water treatment has recently gained extensive attention in academic communities [6].

The rationale behind the photocatalytic approach is based on the in situ generation of highly reactive transient species for the mineralization of refractory organic compounds, ultimately transforming them into harmless carbon dioxide and water. A variety of semiconductor catalysts have demonstrated its efficiency in degrading a wide range of refractory organics, such as TiO_2_ [7], ZnO [8], CdS [9], BaTiO_3_ [10], etc., and the degradation process exhibits excellent performance, operating at ambient pressure and temperature, low cost, and with a lack of secondary waste formation [11]. BaTiO_3_ is considered an efficient semiconductor photocatalyst among various photocatalytic materials due to its perovskite structure, appropriate valance band (VB)/conduction band (CB) positions, availability in a wide variety of sizes and morphologies, environmental friendliness, and good stability [12]. In a typical study, Alammar et al. [13] synthesized BaTiO_3_ nanoparticles by one-step room-temperature ultrasound synthesis in an ionic liquid. The photocatalytic activity of the obtained sample was evaluated by the degradation of methylene blue (MB) under UV irradiation. The removal rate for the MB solution (10 mg/L) was approximately 40% within 180 min, which highlighted the promising application potential of BaTiO_3_ nanoparticles in the field of photocatalytic degradation of organic dyes. Nevertheless, the photocatalytic activity of a general BaTiO_3_ nanoparticle photocatalyst is inherently limited by its low specific surface area [14] and the high recombination rates of photoinduced charged species (electrons and holes) [15]. Surface morphology and size control is a crucial approach for enhancing the photocatalytic efficiency of materials, as it facilitates an increased number of reactive sites. Numerous studies have been dedicated to synthesizing BaTiO_3_-based materials with various morphologies, such as one-dimensional (1D) nanotubes [16], two-dimensional (2D) nanoflowers [17], and three-dimensional (3D) aerogels [18], aiming to enhance their photocatalytic efficiency. Currently, the literature on BaTiO_3_-based materials predominantly focuses on 1D and 2D materials rather than 3D aerogel materials. Despite the exceptional potential of aerogel materials in adsorption and photocatalysis due to their unique surface area and abundant pore structure [19,20,21], the synthesis of BaTiO_3_ aerogel remains a highly intricate challenge that has yet to be fully resolved [18,22]. Chau et al. [18] employed chitin nanocrystals as a liquid crystal template for the synthesis of BaTiO_3_ aerogel. The obtained BaTiO_3_ aerogel exhibited an attenuated specific surface area of only 50 m^2^/g due to the removal of the chitin template at a high temperature (900 °C). The photocatalytic degradation efficiency towards MB exhibited a slight improvement compared to that of P25. Rechberger et al. [22] present a strategy for assembling surface-functionalized nanocrystalline BaTiO_3_ particles into a highly porous macroscopic framework. The gelation mechanism is based on a rapidly induced destabilization of the BaTiO_3_ nanoparticles in dispersion. The bulk aerogel remained stable after supercritical drying and exhibited an unprecedentedly high surface area of over 300 m^2^/g, but the photocatalytic property has not been discussed. The aforementioned strategy, while capable of yielding aerogel samples with a substantial specific surface area, entails a complex synthesis process that is unsuitable for large-scale industrial production. The deposition of noble metals is frequently employed to effectively tackle the critical issue of high recombination rates of photoinduced charged species (electrons and holes). The nanostructured metals can act as electron traps, leading to improvement in the separation of electrons and holes, and consequently enhancing photocatalytic activity [23,24]. Liu et al. [15] successfully prepared the flower-like BaTiO_3_ nanotube arrays (NTAs) by a hydrothermal method using TiO_2_ NTAs as precursors. Ag-loaded BaTiO_3_ NTAs were formed by a photochemical reduction method. The photocatalytic activity of Ag-BaTiO_3_ NTAs was evaluated from the analysis of the photodegradation of methyl orange (MO). It can be seen that silver-modified BaTiO_3_ NTAs have a much higher photocatalytic degradation rate than that of pure BaTiO_3_ NTAs. Loading noble metal nanoparticles onto BaTiO_3_ can establish a Schottky barrier, wherein the Fermi level of the metal nanoparticles is positioned lower than that of the CB of BaTiO_3_. Consequently, photogenerated electrons migrate from the CB of BaTiO_3_ to the metal nanoparticles until their Fermi levels align, resulting in photogenerated holes’ formation within the VB. Thus, spatial separation of photogenerated electron–hole pairs occurs. The noble metal nanoparticles serve as electron traps capable of capturing excited electrons, thereby facilitating efficient separation and reduced recombination of electron–hole pairs [25]. However, the dispersibility of noble metal nanoparticles on the catalyst surface may be constrained due to the small specific surface area of the parent material. Previous studies frequently employed graphene as a support material for loading photocatalysts, aiming to augment the specific surface area and improve the distribution of metal particles on the catalyst surface. Passi et al. [26] designed the Ag-BaTiO_3_/GO nanocomposite, and the results showed that GO nanosheets served as the ground for both photodeposited Ag and BaTiO_3_. To date, research on using a bare catalyst material with a large specific surface area for surface modification by noble metal nanoparticles has been less frequently reported.

Herein, a liquid-phase and template-free synthetic route was employed to obtain a novel BaTiO_3_ aerogel through the co-gelling of metallic alkoxides and supercritical drying without any heat treatment. Subsequently, noble metal Ag nanoparticles were deposited onto the aerogel surface via the photochemical deposition method. The exceptional surface area and abundant pore structure of the as-prepared aerogel photocatalyst provide a great number of active sites, while the surface modification with silver nanoparticles effectively enhances the capture of photogenerated electrons, thereby ensuring an elevated level of photocatalytic efficiency. The preparation process of aerogel solely entails facile agitation and supercritical drying, rendering it highly amenable to industrialization. Furthermore, this method exhibits the potential for broad application in the synthesis of various perovskite aerogels. Consequently, the experimental methodologies exhibit universality, ease of operation, and suitability for large-scale industrial production.

## 2. Results and Discussion

**Figure 1:**
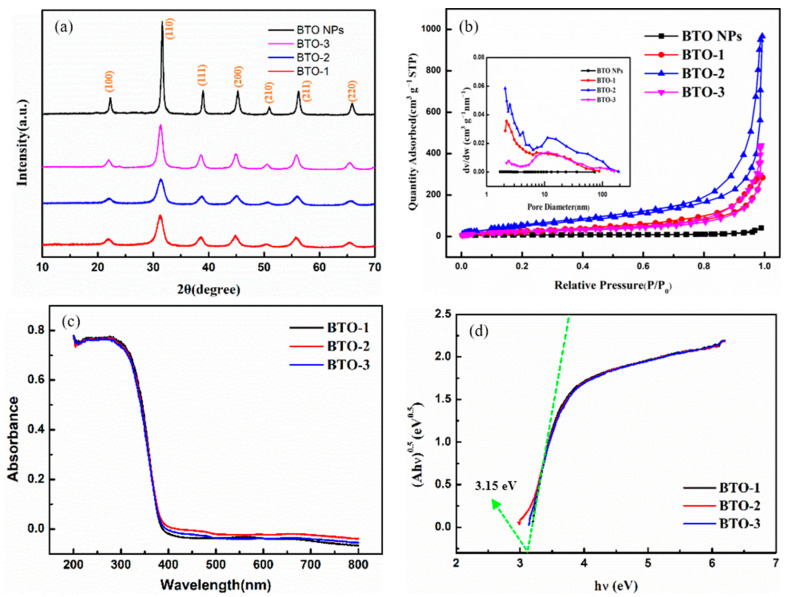
(**a**) XRD patterns of BaTiO_3_ nanoparticles and aerogels. (**b**) Nitrogen adsorption/desorption isotherms of BaTiO_3_ nanoparticles and aerogels; the inset picture is pore-size distribution curves of BaTiO_3_ nanoparticles and aerogels. (**c**) UV-Vis absorption spectra of BaTiO_3_ aerogels. (**d**) (Ahν)^1/2^-hν curve for band gap calculation of BaTiO_3_ aerogels.

The conventional process for synthesizing aerogels involves five steps: sol–gel, solvent exchange, aging, supercritical drying, and heat treatment [27,28]. The BaTiO_3_ nanoparticles (BTO NPs) were synthesized utilizing the aforementioned procedure. The aged gel underwent supercritical drying and calcination, resulting in the formation of crystalline BaTiO_3_. As shown in Figure 1a, the obtained diffraction peaks of BTO NPs positioned at 21.9° (100), 31.3° (110), 38.7° (111), 45.0° (200), 50.7° (210), 55.9° (211), and 65.5° (220) can be well assigned to a cubic phase of BaTiO_3_ (JCPDS 31-0174) [29]. Nitrogen adsorption/desorption studies (Figure 1b and Table 1) show that BTO NPs have a mesoporous structure with a pore size distribution at ~20 nm and a low specific surface area of ~22 m^2^/g. The absence of an aerogel structure in the prepared BTO NPs is evident due to their limited specific surface area and lack of a well-developed pore structure. We assume that the primary impediment to the successful synthesis of BaTiO_3_ aerogels through conventional methods lies in the limited hydrolytic capacity of inorganic salts (Ba^2+^), thereby hindering their involvement in gel network formation (tends to form TiO_2_). Only when the calcination of the gel precursor can lead to the incorporation of Ba^2+^ into TiO_2_ can perovskite BaTiO_3_ crystals be generated. The material undergoes significant shrinkage due to high-temperature calcination, resulting in the deterioration of its pore structure and subsequent failure in aerogel formation. Given the above, we have employed metal alkoxides with hydrolysis rates comparable to that of titanium (IV) isopropoxide to optimize the preparation process. The synthesis of BaTiO_3_ aerogels was accomplished through the co-gelation of metal alkoxides and supercritical drying, eliminating the need for additional heat treatment. The specific preparation process can be found in Section 4 (Materials and Methods). The results are presented in Figure 1 and Table 1, where the as-synthesized BTO-1, BTO-2, and BTO-3 aerogels also exhibit a crystalline cubic phase of BaTiO_3_ (JCPDS 31-0174) and reveal a significantly enhanced specific surface area in comparison to that of BTO NPs. As summarized in Table 1, the specific surface areas of the three aerogels are ~120 m^2^/g (BTO-1), ~220 m^2^/g (BTO-2), and ~90 m^2^/g (BTO-3), respectively. These values are several to even ten times larger than those of BTO NPs. The adsorption/desorption isotherms of all the aerogels are identified as type IV with an H-1 type hysteresis loop, which is a typical characteristic of mesoporous materials [30]. The presence of a three-dimensional continuous mesoporous structure is further supported by the exceptional total pore volume and average pore sizes (Table 1). BTO-2 exhibits a more remarkable porous structure than the other two samples, and its specific surface area is more than four times higher than that of the reported BaTiO_3_ aerogel prepared via the template methods in the literature [18]. Therefore, the modified aerogel procedure offers a more user-friendly and efficient approach to synthesizing BaTiO_3_ aerogels. Since the metal alkoxide is based on the zero-valent metal reacting directly with the alcohol, the modified aerogel procedure demonstrates significant potential for wide-ranging applications in synthesizing various perovskite aerogels. In order to explore the optical properties of BaTiO_3_ aerogel samples, the UV-Vis absorption spectra were recorded. The UV-Vis absorption spectra of BaTiO_3_ aerogel samples were baseline-corrected. In Figure 1c, the absorption spectra of the three aerogel samples exhibit a high degree of similarity, indicating a clear and evident trend. The bandgaps of BTO-1, BTO-2, and BTO-3 samples were further determined by analyzing the (Ahν)^1/2^-hν relationship curve, which was derived using the formula proposed by Tauc, Davis, and Mott et al. The (Ahν)^1/2^-hν curve for bandgap calculation was conducted according to the baseline-corrected absorption spectra. Here, hν was set as the *x*-axis, (Ahν)^1/2^ was set as the *y*-axis, and the reverse extension of the tangent line intersected with the *x*-axis. The intersection on the *x*-axis represents the value of the optical bandgap. As shown in Figure 1d, the calculated bandgaps for all the samples are ~3.2 eV. The results are consistent with those reported in the literature [10,12].

**Figure 2:**
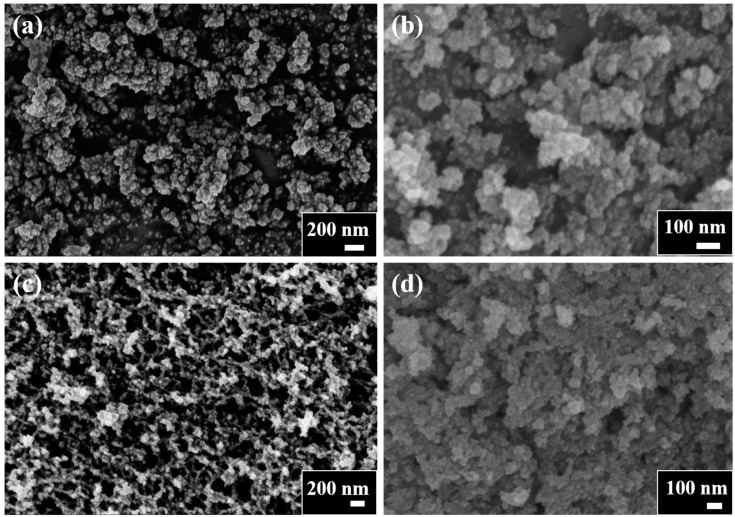
SEM images of (**a**) BTO NPs, (**b**) BTO-1, (**c**) BTO-2, and (**d**) BTO-3 samples.

In the preparation of aerogels, alcohols are frequently selected as solvents to effectively reduce the shrinkage of the materials during gelation and supercritical drying processes. Through extensive and ongoing investigation into the synthesis of aerogels, it has been found that that a combination of alcohol and a hydrophobic solvent promotes the formation of high-quality aerogels. According to the literature [31], the inclusion of toluene in the binary solvent appears to act as a surfactant, which reduces surface tension at the gas–liquid–pore interfaces. As a result, wet gels are formed that have lower density and higher porosity, ultimately resulting in products with larger surface areas. Different alcohols in mixture with toluene lead to aerogels with different properties: micromorphology, specific surface area, pore volume, and pore size distribution. The impact of this binary solvent on the aerogel process necessitates further investigation. Figure 2 shows SEM images of BTO NPs and BaTiO_3_ aerogels. Obviously, BTO NPs present a disordered structure with an irregular arrangement of nanoparticles, as shown in Figure 2a. Among all the three aerogel samples, only the BTO-2 sample exhibits a continuous three-dimensional network skeleton, as shown in Figure 2c. The distinct three-dimensional network skeleton structure is not observed in BTO-1 and BTO-3 samples, while the micromorphology appears to exhibit particle agglomeration, as shown in Figure 2b,d. The aforementioned findings are in line with the data presented in Table 1. In conclusion, the ethanol and toluene mixture emerges as the optimal solvent for synthesizing BaTiO_3_ aerogel, with superior characteristics in terms of specific surface area and pore structure. In contrast to the conventional sol–gel approach used for synthesizing BaTiO_3_ NPs, barium ethylate can participate in gel network formation through hydrolysis. During the initial stages of gelation in this system, a Ti-rich oxide network is formed, accompanied by a significant presence of unreacted Ba ions within the liquid filling the pores due to the high solubility of Ba(OH)_2_ (formed by hydrolysis) in the ethanol and toluene mixture solvent. The Ba(OH)_2_ in the ethanol and toluene mixture solvent subsequently undergoes co-gelation, leading to the formation of chemical bonds within the gel network and resulting in the crystalline BaTiO_3_ particulate gel. The bonding between the Ti and Ba species is speculated to involve a repeating unit of (-Ti-O-)_n_Ba^2+^, according to the literature [32]. Therefore, the corresponding aerogel can be obtained by supercritical drying of the crystalline BaTiO_3_ particulate gel without additional heat treatment. Although further investigation has been conducted on the impact of binary solvents on the aerogel process, regrettably, due to the insufficient crucial data and experimental evidence, a clear mechanism elucidating why ethanol–toluene in the binary solvents is more suitable for BaTiO_3_ aerogel preparation than methanol–toluene and isopropanol–toluene remains elusive.

**Figure 3:**
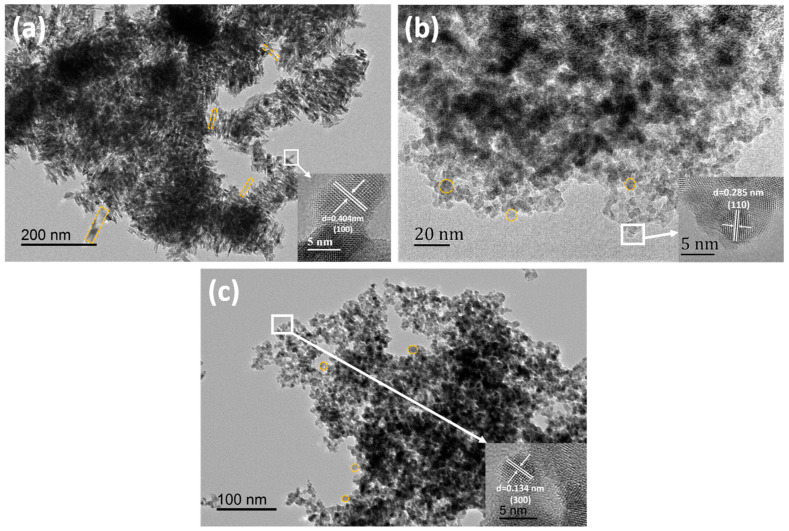
TEM micrographs of (**a**) BTO-1, (**b**) BTO-2, and (**c**) BTO-3 aerogel samples (HRTEM micrographs inset).

The micromorphology was further studied by transmission electron microscopy (TEM). Figure 3 shows TEM micrographs of BTO-1, BTO-2, and BTO-3 aerogel samples. The BTO-1 aerogel sample exhibits a morphology characterized by short rod-shaped particles (Figure 3a) while BTO-2 and BTO-3 aerogel samples exhibit a morphology characterized by irregular spherical particles (Figure 3b,c). Evidently, the directional growth of BaTiO_3_ grains is constrained by the binary solvent system comprising methanol and toluene. Surface morphology and size control is a crucial approach for enhancing the photocatalytic efficiency of materials. The impact of micromorphology on photocatalytic efficiency is further investigated in the subsequent photodegradation experiments. The high-resolution TEM (HRTEM) micrographs further prove that the lattice stripe spacing is 0.404 nm (inset in Figure 3a), 0.285 nm (inset in Figure 3b), and 0.134 nm (inset in Figure 3c), corresponding to the (100), (110), and (300) crystal planes of the cubic phase BaTiO_3_, respectively. The results are consistent with the XRD spectrum (Figure 1a).

**Figure 4:**
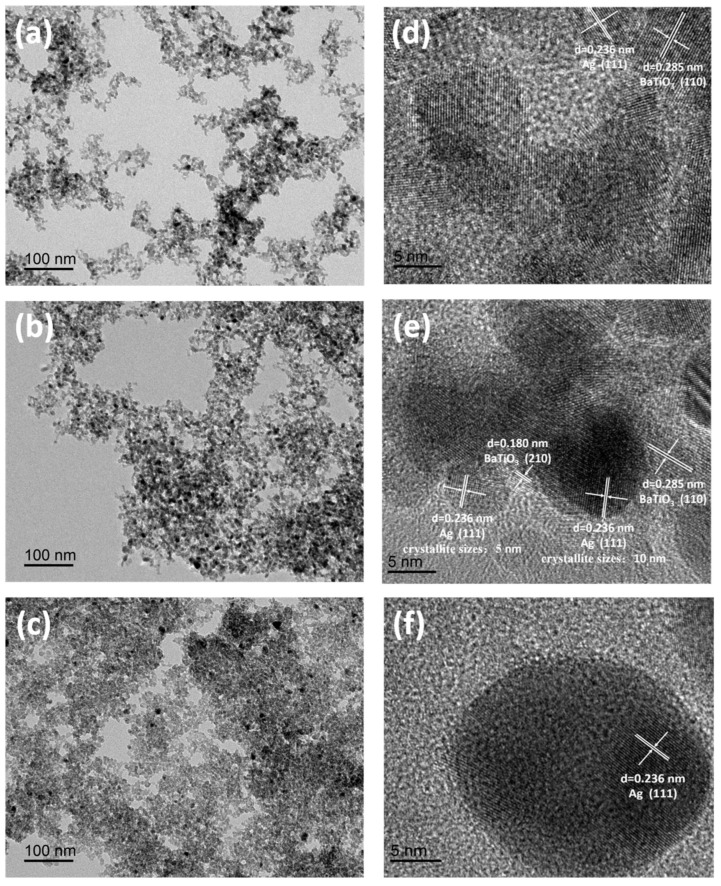
TEM images of 1% Ag/BTO-2 (**a**), 3% Ag/BTO-2 (**b**), and 5% Ag/BTO-2 (**c**) samples. HRTEM images of 1% Ag/BTO-2 (**d**), 3% Ag/BTO-2 (**e**), and 5% Ag/BTO-2 (**f**) samples.

The BTO-2 aerogel sample synthesized by using an ethanol–toluene binary solvent exhibits superior microstructure and pore structure, which is a very suitable method for Ag nanoparticles’ surface modification in the next work. Figure 4 shows TEM and HRTEM micrographs of BTO-2 aerogel samples with varying amounts of Ag deposition. It is evident in Figure 4a–c that Ag nanoparticles are uniformly distributed on BTO-2 aerogel in all modified samples. Upon increasing the Ag deposition amount from 1% to 5%, a noticeable augmentation in the size of Ag nanoparticles is observed, from several nanometers to dozens of nanometers (Figure 4d–f). Furthermore, the size differences of Ag nanoparticles grown on different exposed crystal planes can also be observed. The dimension of Ag nanoparticles on the (100) crystal plane of BaTiO_3_ in the 3% Ag/BTO-2 sample shown in Figure 4e is approximately 10 nm, while that on the (210) crystal plane measures around 5 nm. The observed phenomenon suggests that the prepared BaTiO_3_ aerogel exhibits a polycrystalline nature, with distinct crystal planes exposed. Some of these crystal planes demonstrate enhanced suitability for Ag nanoparticle growth, while others exhibit slower rates of Ag nanoparticle formation. Works of literature [33,34] have revealed that the phenomenon is primarily associated with the surface energy of the exposed crystal plane. High-energy surface atoms exhibit high activity and are easy to combine with foreign atoms such as the Ag or Au atoms to form a stable structure and significantly increase the number of loading noble metal nanoparticles on the material surface. The impact of different Ag deposition amounts on photocatalytic efficiency is further investigated in the subsequent photodegradation experiments. 

**Figure 5:**
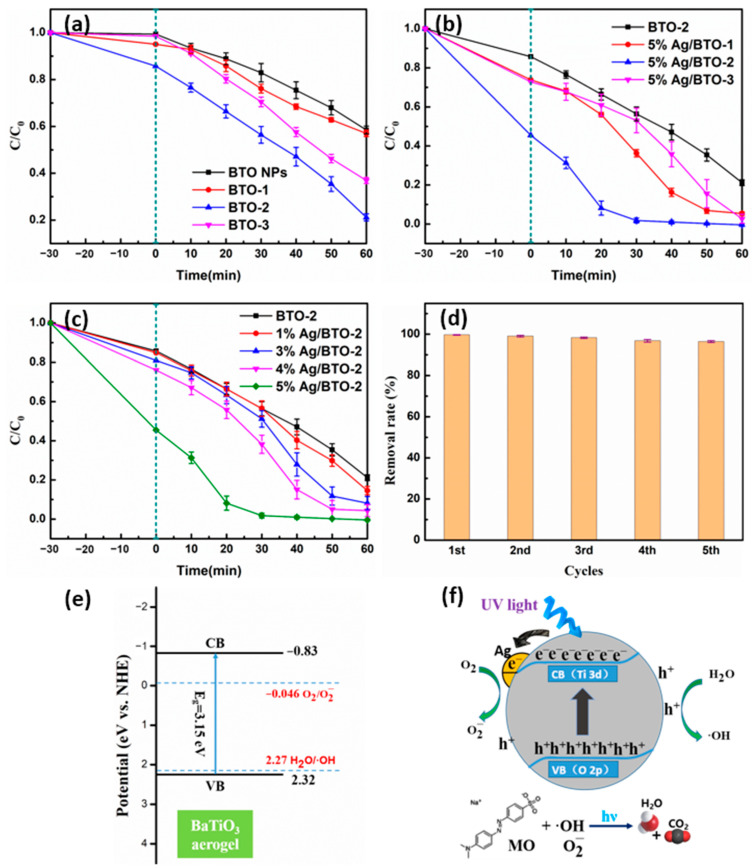
The photocatalytic degradation curve of MO by BaTiO_3_ nanoparticles and aerogels (**a**), BTO-2 aerogel and 5 wt% Ag nanoparticles deposited onto BaTiO_3_ aerogels (**b**), and BTO-2 aerogel and varying Ag nanoparticles deposited onto BTO-2 aerogels (**c**). The cycling degradation efficiency of 5% Ag/BTO-2 under UV light irradiation (**d**). The energy diagram with the valence and the conduction bands of BaTiO_3_ and the energies of O_2_/$O_{2}^{-}$ and H_2_O/·OH couples (**e**). Schematic illustration of photodegradation process of Ag nanoparticles deposited onto BaTiO_3_ aerogels (**f**).

The degradation of MO by BTO NPs, BTO-1, BTO-2, and BTO-3 aerogel under ultraviolet light irradiation was studied. In a typical adsorption experiment, we investigated the adsorption of aerogel of MO over a longer period of time (60 min) and observed consistent equilibrium adsorption amounts at both 30 min and 60 min. The C/C_0_ values at 30 min and after 1 h of stirring in the dark are shown in Table 2. The results indicate that the adsorption–desorption equilibrium of the MO and the aerogel can be established in 30 min. To strike a balance between experimental efficiency and achieving the complete establishment of the adsorption–desorption equilibrium, we determined the optimal adsorption time to be 30 min. As shown in Figure 5a, after the adsorption equilibrium is reached in the dark (although Figure 5a–c only provide the C/C_0_ value at the adsorption endpoint, it can be determined that the adsorption–desorption equilibrium has been reached), the removal efficiency (adsorption) of all BaTiO_3_ aerogel samples of MO surpasses that of bare BaTiO_3_ NPs, owing to their porous structure. Specifically, the adsorption efficiency of BaTiO3 NPs was found to be less than 1%, whereas the three aerogel samples exhibited adsorption efficiencies of 5% (BTO-1), 14% (BTO-2), and 2% (BTO-3), respectively. There is no doubt that the three-dimensional continuous porous structure of BaTiO_3_ aerogels provides a convenient transfer channel for the adsorption of MO. The BTO-2 aerogel sample exhibited superior adsorption efficiency, attributed to the exceptional specific surface area and total pore volume, whereas the adsorption efficiency of BTO-1 and BTO-3 samples was only marginally higher than that of BTO NPs due to their inadequate specific surface area and total pore volume. When it comes to photocatalysis after ultraviolet light irradiation, the removal efficiency of all BaTiO_3_ aerogel samples was also higher than that of bare BaTiO_3_ NPs. It is noteworthy that despite BTO-1 exhibiting a larger specific surface area and total pore volume compared to BTO NPs, the removal efficiencies of both samples were remarkably similar, with 43% for the former and 42% for the latter. Possible reasons include the potential impact of micromorphology, as nanorod-shaped particles (BTO-1) may exhibit lower photocatalytic efficiency compared to spherical particles (BTO-2, BTO-3, and even BTO NPs). Similar results were reported in the literature [17], demonstrating that the photocatalytic activity of the BaTiO_3_ material with square particles was higher compared to spherical nanoflowers and short rods. The removal efficiency of the BTO-2 aerogel sample for MO was almost 80% after 60 min of illumination, which is nearly twice as high as that of the BTO NPs sample. On the one hand, the three-dimensional continuous porous structure of aerogel provides a convenient transfer channel for the in situ degradation of MO [35]. On the other hand, it facilitates an increased number of reactive sites [36,37]. Therefore, the BTO-2 sample exhibits enhanced photocatalytic efficiency. In order to further improve the photocatalytic efficiency of the sample and increase its commercial value, we also studied the effect of the introduction of noble metal Ag nanoparticles on the performance of the samples. As shown in Figure 5b, the surface modification with Ag nanoparticles effectively enhanced the MO removal efficiency. MO was almost completely degraded by the Ag nanoparticles deposited on the BaTiO_3_ aerogel samples after 60 min of illumination, while the optimum removal efficiency of the BaTiO_3_ aerogel samples was 80% compared with the unmodified sample. The introduction of noble metal Ag nanoparticles onto BaTiO_3_ leads to the spatial separation of photogenerated electron–hole pairs through a Schottky barrier [25]. As a result, it contributes to the enhanced photocatalytic activity of Ag nanoparticles deposited onto BaTiO_3_ aerogel. A similar trend of enhancement was observed in the adsorption efficiency of MO. The three Ag nanoparticles deposited on the BaTiO_3_ aerogel samples exhibited adsorption efficiencies of 26% (5% Ag/BTO-1), 54% (5% Ag/BTO-2), and 27% (5% Ag/BTO-3), respectively. The enhanced adsorption of dye molecules by noble metals can be attributed to the potential alteration in the electronic state of the substrate resulting from the deposition of noble metal nanoparticles on its surface [23]. In acidic media of pH below 6.5, BaTiO_3_ surfaces are positively charged, while MO is negatively charged [38,39]. Due to the strong electrostatic attraction between these charged species, the facile adsorption of MO on the surface of BaTiO_3_ can be observed. The interaction between Ag and BaTiO_3_ may potentially alter the electronic state of BaTiO_3_ by the possible transferred charge carriers, thereby resulting in an enhanced adsorption phenomenon. Relevant occurrences have previously been documented [40,41]. However, the previously mentioned photocatalyst was constrained by its limited specific surface area, and it exhibited a slight increase in adsorptive capacity. The adsorption capacity was significantly enhanced when using a bare catalyst material with a large specific surface area for surface modification by metal nanoparticles. This may be attributed to the fact that the substrate material, which has a larger specific surface area, provides the necessary conditions for achieving uniform dispersion of Ag on its surface. As the sample with the highest adsorption and photocatalytic efficiency, BTO-2 was further studied for its photocatalytic performance with different Ag deposition amounts. The results are shown in Figure 5c. Evidently, the augmentation of Ag deposition quantity leads to varying degrees of enhancement in both the adsorption efficiency and photocatalytic efficiency of the modified BTO-2 aerogel samples. The Ag nanoparticles deposited on the BTO-2 aerogel samples exhibited adsorption efficiencies of 15% (1% Ag/BTO-2), 19% (3% Ag/BTO-2), 24% (4% Ag/BTO-2), and 54% (5% Ag/BTO-2), respectively. The size of Ag nanoparticles exhibited a positive correlation with the deposition amount (Figure 4). In general, larger Ag nanoparticles exhibited an enhanced adsorption capacity for MO due to the potential alteration in the electronic state of the substrate resulting from the deposition of noble metal nanoparticles on its surface, and they exhibited higher photocatalytic efficiency due to the spatial separation of photogenerated electron–hole pairs through a Schottky barrier.

To further investigate the photocatalytic activities of BTO NPs, BaTiO_3_ aerogel samples, and Ag nanoparticles deposited onto BaTiO_3_ aerogel samples, pseudo-first-order kinetics were employed to analyze the photocatalytic degradation kinetics. The kinetics of photocatalytic degradation of MO solution were calculated from the simplified form of the Langmuir–Hinshelwood model, which can be described as Equation (1):

(1) $$ln\frac{C_{0}}{C}=kt$$

where *C*_0_ is the initial concentration (mg/L) of the MO solution and *C* is the concentration (mg/L) of the MO solution at any time *t* (min), and *k* is the pseudo-first-order rate constant (min^−1^). Obviously, the degradation rate *k* can be obtained from the slope of *ln*(*C*_0_/*C*) vs. *t* and the time *t*_1/2_ required to degrade half of the MO solution can be calculated by *ln*2/*k*. The calculation results of various dynamics parameters have been summarized and presented in Table 3. The squared correlation coefficient (*R*^2^) falls within the range of 91.44–99.67%, indicating the reliability of the fitting curve for the pseudo-first-order kinetic equation. The photocatalytic degradation of MO catalyzed by the 5% Ag/BTO-2 aerogel sample exhibited the highest reaction rate, with *k* = 0.1181 min^−1^, which was almost 4.9-fold higher than that of the initial BTO-2 sample (*k* = 0.0243 min^−1^) and 13.7-fold higher than that of the BTO NPs (*k* = 0.0086 min^−1^). Table 4 lists the photocatalytic performance of various catalysts for the degradation of MO. The BaTiO_3_ aerogel in this work (BTO-2) exhibits enhanced photocatalytic degradation performance compared to the traditional BaTiO_3_ nanomaterials and commercialized P25. The use of a bare BaTiO_3_ aerogel with a large specific surface area as the ground for photodeposited Ag nanoparticles has also achieved impressive results. The 5% Ag/BTO-2 aerogel exhibits superior or comparable photocatalytic degradation performance to other BaTiO_3_ composite photocatalysts. Thus, the utilization of material modification techniques based on high-performance photocatalysts with exceptional specific surface areas presents unparalleled advantages in augmenting the capabilities and broadening the scope of photocatalysts for future applications. The recycling and long-term stability of catalysts are pivotal attributes for practical applications. The stability and reusability of the Ag nanoparticles deposited onto the BaTiO_3_ aerogel photocatalysts were further evaluated through additional experiments, as depicted in Figure 5d. After five cycles of photocatalytic degradation of MO under identical experimental conditions, the 5% Ag/BTO-2 aerogel exhibited a remarkable preservation rate of 96.4% for the removal efficiency. Furthermore, the aerogel can be effectively recycled for subsequent cycles through a straightforward filtration and washing process prior to each cycle. The error in each experimental group was exceptionally small, indicating the robustness of the synthesis process and the consistent performance of the material.

In order to comprehend the photocatalytic mechanism of BaTiO_3_ aerogels, it is imperative to determine their energy-band potentials, as the redox ability of photogenerated electrons and holes is closely associated with these potentials. The VB and CB potentials of BaTiO_3_ can be calculated by Equations (2) and (3) [42,43]:

(2) $$E_{VB}=\chi -E^{e}+0.5E_{g}$$

(3) $$E_{CB}=\chi -E^{e}-0.5E_{g}$$

where *E_VB_* and *E_CB_* are the VB edge and CB edge potentials (eV vs. NHE), *χ* is the absolute electronegativity of the BaTiO_3_ aerogel. The term is defined as the arithmetic mean of the electron affinity and the first ionization energy of the constituent atoms and can be calculated to be 5.25 eV, according to the literature [44]. *E^e^* is the energy of free electrons on the hydrogen scale (~4.5 eV). The bandgap energy *E_g_* of BaTiO_3_ aerogel is 3.15 eV, as shown in Figure 1d. As a result, the VB edge and CB edge potentials are estimated to be 2.32 and −0.83 eV vs. the normal hydrogen electrode (NHE), respectively. Figure 5e shows an energy diagram with the valence and the conduction bands of BaTiO_3_ and the energies of O_2_/$O_{2}^{-}$ and H_2_O/·OH couples. It is clearly visible that the *E_VB_* of BaTiO_3_ aerogel is more positive than the redox potential of H_2_O/·OH (2.27 eV vs. NHE) and the *E_CB_* of BaTiO_3_ aerogel is more negative than the standard redox potential of O_2_/$O_{2}^{-}$ (−0.046 eV vs. NHE) [45], resulting in the formation of a large amount of $O_{2}^{-}$ and ·OH radicals. The schematic diagram depicting the photocatalytic mechanisms of Ag nanoparticles deposited onto BaTiO_3_ aerogel is presented in Figure 5f. Under ultraviolet light, photogenerated electrons and holes are produced in the 5% Ag/BTO-2 sample. The photogenerated electrons transfer from the O 2p orbital (VB) to the Ti 3d orbital (CB), subsequently migrating towards the metallic Ag and accumulating on its surface. These electrons can be rapidly transferred to the adsorbed oxygen on the Ag surface, resulting in the generation of reactive oxygen species ($O_{2}^{-}$). Concurrently, the photogenerated holes can efficiently transfer to the aerogel surface, leading to the generation of highly reactive ·OH radicals. Both ·OH radicals and $O_{2}^{-}$ radicals possess potent oxidizing capabilities, facilitating the complete oxidation of MO into H_2_O and CO_2_. Acting as photogenerated electron traps, Ag nanoparticles enhance the rate of electron transfer to molecular oxygen and suppresses the recombination of photogenerated electrons and holes. Meanwhile, the three-dimensional porous structure of the BaTiO_3_ aerogel provides suitable transport channels for adsorption and photocatalysis and generates more active sites. As a result, these structure and components contribute to the enhanced photocatalytic activity of Ag nanoparticles deposited onto BaTiO_3_ aerogel.

**Table 2:** The C/C_0_ values at 30 min and after 1 h of stirring in the dark.

Adsorption Time (min)	C/C_0_ (%)									
	BTO NPs	BTO-1	BTO-2	BTO-3	5% Ag/ BTO-1	5% Ag/ BTO-2	5% Ag/ BTO-3	1% Ag/ BTO-2	3% Ag/ BTO-2	4% Ag/ BTO-2
30	99.37	95.06	85.75	98.47	73.88	45.51	72.76	84.87	80.94	76.01
60	99.35	95.10	85.83	98.44	73.96	46.02	72.68	84.64	81.06	75.97

**Table 3:** The various calculated dynamics parameters for photodegradation.

Sample	k (min^−1^)	t_1/2_ (min)	R^2^ (%)
BTO NPs	0.0086	80.6	96.08
BTO-1	0.0099	70.0	99.67
BTO-2	0.0243	28.5	91.44
BTO-3	0.0182	38.1	98.09
5% Ag/BTO-1	0.0518	13.4	92.30
5% Ag/BTO-2	0.1181	5.9	98.96
5% Ag/BTO-3	0.0521	13.3	95.32

**Table 4:** Photocatalytic performance of various catalysts for the degradation of MO.

Photocatalyst	Morphology	Photocatalytic Performance	Photodegradation Kinetics	Ref
BTO-2 (1g/L)	Aerogels	80% for MO, 60 min (10 mg/L, 100 mL)	0.0243 min^−1^	This work
BTO-NPs (1g/L)	Irregular NPs	42% for MO, 60 min (10 mg/L, 100 mL)	0.0086 min^−1^	This work
BaTiO_3_ (—)	Coral cluster	65% for MO, 150 min (Not mentioned)	0.0074 min^−1^	[46]
BaTiO_3_ (—)	Nanocube	76% for MO, 45 min (20 mg/L, 60 mL)	Not mentioned	[47]
P25 (0.04 g)	Irregular NPs	~52% for MO, 90 min (20 mg/L, 150 mL)	Not mentioned	[48]
5% Ag/BTO-2 (1g/L)	Aerogels	99% for MO, 40 min (10 mg/L, 100 mL)	0.1181 min^−1^	This work
Ag/BaTiO_3_ (6 cm^2^)	Nanotube and flower	98% for MO, 60 min (20 mg/L, 75 mL)	0.0707 min^−1^	[15]
BaTiO_3_@g-C_3_N_4_ (0.5 g/L)	Irregular NPs	76% for MO, 360 min (5 mg/L)	Not mentioned	[43]
BaTiO_3_/In_2_S_3_ (0.5 g/L)	Core–shell	93% for MO, 90 min (10 mg/L, 100 mL)	0.0334 min^−1^	[49]
Bi_2_O_3_/BaTiO_3_ (2 g/L)	Irregular NPs	99% for MO, 50 min (10 mg/L, 300 mL)	0.1100 min^−1^	[14]
BaTiO_3_/rGO (0.05 g)	Nanosheet and NPs	70% for MO, 20 min (0.05 mM, 50 mL)	0.0556 min^−1^	[50]

## 3. Conclusions

In this work, BaTiO_3_ aerogels were prepared via a liquid-phase and template-free method, eliminating the necessity for thermal treatment. The crystalline BaTiO_3_ aerogels can be directly obtained through co-gelation and supercritical drying processes following the hydrolysis of metal alkoxides. The BTO-2 aerogel sample was prepared using a mixed solvent of ethanol and toluene, and the conclusion is summarized as follows:

(1) The utilization of a blend comprising alcohols and hydrophobic solvents demonstrates effective mitigation of material shrinkage during gelation and supercritical drying.
(2) The prepared BTO-2 aerogel exhibits superior specific surface area (223 m^2^/g) and total pore volume (1.50 cm^3^/g), and thus has the best photocatalytic degradation performance.
(3) Noble metal Ag nanoparticles’ deposition onto the BaTiO_3_ aerogel surface enhances the capture of photogenerated electrons, thereby ensuring an enhanced property of photocatalytic efficiency.
(4) After Ag nanoparticles were deposited on BaTiO_3_ aerogel, BaTiO_3_ aerogel could degrade MO completely after 40 min of illumination.

The recoverability and long-term stability of the aerogel photocatalysts have also been demonstrated to be reliable.

The preparation process of aerogel in this work solely entails facile agitation and supercritical drying, rendering it highly amenable to industrialization.

## 4. Materials and Methods

### 4.1. Materials

Titanium (IV) isopropoxide (C_12_H_28_O_4_Ti, 95%), acetic acid (CH_3_COOH, 99.5%), barium metal (Ba, 99%), and methyl orange (C_14_H_14_N_3_SO_3_Na, 96%) were purchased from Aladdin Chemistry Co., Ltd. (Shanghai, China). Ethanol (CH_3_CH_2_OH, 99.7%) was supplied by Yasheng Chemical Co., Ltd. (Wuxi, China). Tetrabutyl titanate (C_16_H_36_O_4_Ti, 98%) and toluene (C_7_H_8_, 99.7%) were purchased from Lingfeng Chemical Reagent Co., Ltd. (Shanghai, China). Barium acetate ((CH_3_COO)_2_Ba, 99%), methanol (CH_3_OH, 99.5%), isopropanol ((CH_3_)_2_CH_2_OH, 99.7%), and silver nitrate (AgNO_3_, 99.8%) were purchased from Sinopharm Chemical Reagent Co., Ltd. (Shanghai, China). Deionized water was used during the experiment.

### 4.2. Fabrication of BaTiO_3_ Nanoparticles

An amount of 0.04 mol of barium acetate was added to the mixture of acetic acid (20 mL) and deionized water (20 mL) and dissolved by stirring at room temperature, called Solution A. Meanwhile, 0.04 mol of tetrabutyl titanate was added to 40 mL of ethanol and dispersed by stirring at room temperature, called Solution B. The dropwise addition of Solution B to Solution A was followed by stirring for 0.5 h until complete hydrolysis occurred, resulting in the formation of a transparent Sol C. After that, Sol C was subjected to gelation by placing it in an oven at a temperature of 50 °C for approximately 1 h, leading to the formation of Gel D. The solvent in Gel D was periodically replaced with ethanol, with each replacement occurring every 24 h. During this period, Gel D was aged in an oven at a temperature of 50 °C for 3 d. Finally, Gel D was dried under supercritical C_2_H_5_OH (10 MPa, 270 °C) conditions for 2 h and the obtained powder sample was calcined at 600 °C for 2 h to prepare BaTiO_3_ nanoparticles, named BTO NPs.

### 4.3. Fabrication of BaTiO_3_ Aerogels

In a typical synthesis, 6 mmol of barium metal (the oxide film of barium metal must be eliminated prior to the experiment) was dissolved in 25 mL of alcohol (methanol, ethanol, or isopropanol) to generate a solution of metal alkoxide. Then, 6 mmol of titanium isopropoxide and 35 mL of toluene were added and the mixture was stirred in a sealed container for 0.5 h until uniform and stable clear solutions were formed (colorless for that prepared by methanol and ethanol, red brown for that prepared by isopropanol, respectively). Subsequently, hydrolysis was induced by adding 0.4 g of deionized water. The resulting solution was left at room temperature with continuous stirring for at least 12 h until it transformed into a slightly milky wet gel (white for that prepared by methanol and ethanol, yellow brown for that prepared by isopropanol, respectively). Finally, the mixture was transferred into four glass bottles with a capacity of 20 mL each and dried under supercritical C_2_H_5_OH (10 MPa, 270 °C) conditions for 2 h to produce BaTiO_3_ aerogels (white for all samples). The obtained BaTiO_3_ aerogels were designated as BTO-1 (prepared by methanol), BTO-2 (prepared by ethanol), and BTO-3 (prepared by isopropanol), respectively.

### 4.4. Ag Nanoparticles’ Deposition on Aerogel Surface

The noble metal Ag nanoparticles were deposited on the aerogel surface via the photochemical deposition method [51]. An amount of 100 mg of the obtained aerogel was added to 10 mL of a solution of AgNO_3_ with a certain concentration (0.001 M, 0.003 M, and 0.005 M), and then continuously stirred under a 500 W ultraviolet lamp for 1 h for photochemical deposition. The deposited samples were collected and washed three times with deionized water at 5000 rpm using centrifugation to remove any residual solution, followed by drying at a temperature of 80 °C for 1 h. According to the calculated amount of approximate Ag deposition (x wt%), the samples were named x% Ag/BTO-y, whose type of the aerogel was y.

### 4.5. Characterization

The crystal structure analysis of the aerogels was carried out on an X-ray powder diffractometer (XRD, SmartLab 3000, Rigaku, Tokyo, Japan) with Cu Kα radiation (λ = 0.15406 nm) and a scanning range of 10–70° at room temperature, and the tube voltage and current of the instrument were operated at 40 kV and 30 mA, respectively. A field emission scanning electron microscope (SEM, LEO-1530VP, LEO/Zeiss, Oberkochen, Germany) and a transmission electron microscope (TEM, JEM-2100, JEOL, Tokyo, Japan) were employed to observe the microscopic morphology and structure of the samples. The Brunauer–Emmett–Teller (BET) specific surface areas, pore volume, and pore distribution were measured by nitrogen adsorption/desorption isotherms using a surface area and pore size analyzer (ASAP2020, Micromeritics, Norcross, GA, USA) after the samples were degassed in a vacuum at 120 °C for 8 h. With a white BaSO_4_ disk as the background, the UV-Vis absorption spectra of samples were recorded on a spectrophotometer (CARY 300, Agilent, Palo Alto, CA, USA) via integrating sphere mode. X-ray photoelectron spectroscopy (XPS, EscaLab 250Xi, Thermo Fisher, Waltham, MA, USA) was used to determine the valence states of the elements on the sample surface. The binding energies were calibrated using containment carbon (C 1s = 284.8 eV).

### 4.6. Photocatalytic Experiment

The photocatalytic activity of the BaTiO_3_ aerogels was measured by the photodegradation of a MO solution under the illumination of ultraviolet light at ambient temperature. In a typical photocatalytic experiment, 100 mg of the BaTiO_3_ aerogel was dispersed in 100 mL of MO solution at a concentration of 10 mg/L, followed by pH adjustment to 3 using 1 M HCl. Before illumination, the mixture was magnetically stirred at 150 rpm for 0.5 h in the dark to establish the adsorption–desorption equilibrium of the MO and the aerogel. The light source utilized in this study was a 500 W mercury lamp. During the photocatalysis, a 5 mL suspension was extracted every 10 min and subsequently centrifuged at a speed of 5000 rpm for 10 min, and then the resulting clear solution was collected. The concentration of the collected clear solution was determined by measuring the absorbance of MO at 463 nm by a 722S UV–visible spectrophotometer. Finally, the photocatalytic efficiency could be calculated via Equation (4):

η = C/C_0_ (4)

where *C*_0_ is the initial concentration (mg/L) of the MO solution and *C* is the concentration (mg/L) of the MO solution at any time. The photocatalytic experiments were conducted in triplicate for each sample, and the average values along with the corresponding errors (standard deviation) were determined.

## Figures and Tables

**Table 1:** The characteristic properties of BaTiO_3_ nanoparticles and aerogels.

Sample	Surface Area (m^2^/g)	Total Pore Volume (cm^3^/g)	Average Pore Sizes (nm)
BTO NPs	21.8	0.06	19.9
BTO-1	119.6	0.44	10.3
BTO-2	223.1	1.50	19.0
BTO-3	89.6	0.68	23.4

## Data Availability

The raw/processed data required to reproduce these findings cannot be shared at this time as the data also form part of an ongoing study.

## References

[1] Khan W.U., Ahmed S., Dhoble Y., Madhav S. (2023). A critical review of hazardous waste generation from textile industries and associated ecological impacts. J. Indian Chem. Soc..

[2] Stella F., Garofalo S.F., Cavallini N., Fino D., Deorsola F.A. (2024). Closing the loop: Analysis of biotechnological processes for sustainable valorisation of textile waste from the fast fashion industry. Sustain. Chem. Pharm..

[3] Kolya H., Kang C.-W. (2024). Toxicity of Metal Oxides, Dyes, and Dissolved Organic Matter in Water: Implications for the Environment and Human Health. Toxics.

[4] Varadarajan G., Venkatachalam P. (2016). Sustainable textile dyeing processes. Environ. Chem. Lett..

[5] Khataee A., Kasiri M.B. (2010). Photocatalytic degradation of organic dyes in the presence of nanostructured titanium dioxide: Influence of the chemical structure of dyes. J. Mol. Catal. A Chem..

[6] Chong M.N., Jin B., Chow C.W., Saint C. (2010). Recent developments in photocatalytic water treatment technology: A review. Water Res..

[7] Lee S.-Y., Park S.-J. (2013). TiO_2_ photocatalyst for water treatment applications. J. Ind. Eng. Chem..

[8] Mylsamy S., Govindasamy T., Subramanian B. (2024). Systematic exploration of defect-rich 2D nanopetal assembled 3D ZnO nanoflowers for improved photocurrent generation and photocatalytic performance. J. Environ. Chem. Eng..

[9] Sun Q., Wang N., Yu J., Yu J.C. (2018). A hollow porous CdS photocatalyst. Adv. Mater..

[10] Kappadan S., Gebreab T.W., Thomas S., Kalarikkal N. (2016). Tetragonal BaTiO_3_ nanoparticles: An efficient photocatalyst for the degradation of organic pollutants. Mat. Sci. Semicon. Proc..

[11] Al-Nuaim M.A., Alwasiti A.A., Shnain Z.Y. (2023). The photocatalytic process in the treatment of polluted water. Chem. Pap..

[12] Ray S.K., Cho J., Hur J. (2021). A critical review on strategies for improving efficiency of BaTiO_3_-based photocatalysts for wastewater treatment. J. Environ. Manag..

[13] Alammar T., Hamm I., Wark M., Mudring A.-V. (2015). Low-temperature route to metal titanate perovskite nanoparticles for photocatalytic applications. Appl. Catal. B Env..

[14] Lin X., Xing J., Wang W., Shan Z., Xu F., Huang F. (2007). Photocatalytic activities of heterojunction semiconductors Bi_2_O_3_/BaTiO_3_: A strategy for the design of efficient combined photocatalysts. J. Phys. Chem. C.

[15] Liu J., Sun Y., Li Z. (2012). Ag loaded flower-like BaTiO_3_ nanotube arrays: Fabrication and enhanced photocatalytic property. CrystEngComm.

[16] Nageri M., Kumar V. (2018). Manganese-doped BaTiO_3_ nanotube arrays for enhanced visible light photocatalytic applications. Mater. Chem. Phys..

[17] Guo Q., Gao T., Padervand M., Du D., Zhao K., Zhang Y., Jia T., Wang C. (2023). Piezo-Photocatalytic Degradation of Tetracycline by 3D BaTiO_3_ Nanomaterials: The Effect of Crystal Structure and Catalyst Loadings. Processes.

[18] Chau T.T.L., Le D.Q.T., Le H.T., Nguyen C.D., Nguyen L.V., Nguyen T.-D. (2017). Chitin liquid-crystal-templated oxide semiconductor aerogels. ACS Appl. Mater. Interfaces.

[19] Yang X.-C., Zhao J.-T. (2024). Aerogel for Highly Efficient Photocatalytic Degradation. Gels.

[20] Li P., Yang C., Xu X., Miao C., He T., Jiang B., Wu W. (2022). Preparation of bio-based aerogel and its adsorption properties for organic dyes. Gels.

[21] Liu J., Wang X., Shi F., Yu L., Liu S., Hu S., Liu D. (2016). Synthesis of mesoporous SiO_2_ aerogel/W_x_TiO_2_ nanocomposites with high adsorptivity and photocatalytic activity. Adv. Powder Technol..

[22] Rechberger F., Heiligtag F.J., Süess M.J., Niederberger M. (2014). Assembly of BaTiO_3_ nanocrystals into macroscopic aerogel monoliths with high surface area. Angew. Chem. Int. Ed..

[23] Cui Y., Briscoe J., Dunn S. (2013). Effect of Ferroelectricity on Solar-Light-Driven Photocatalytic Activity of BaTiO_3_—Influence on the Carrier Separation and Stern Layer Formation. Chem. Mater..

[24] Meng H., Chen Z., Lu Z., Wang X. (2023). Piezoelectric effect enhanced plasmonic photocatalysis in the Pt/BaTiO_3_ heterojunctions. J. Mol. Liq..

[25] Ali M., Swami P., Kumar A., Guin D., Tripathi C.S.P. (2024). Enhanced photocatalytic degradation of Rhodamine B using gold nanoparticles decorated on BaTiO_3_ with surface plasmon resonance enhancement. Anal. Sci..

[26] Passi M., Pal B. (2023). Design of a novel Ag-BaTiO_3_/GO ternary nanocomposite with enhanced visible-light driven photocatalytic performance towards mitigation of carcinogenic organic pollutants. Sep. Purif. Technol..

[27] Du A., Zhou B., Zhang Z., Shen J. (2013). A special material or a new state of matter: A review and reconsideration of the aerogel. Materials.

[28] Niculescu A.-G., Tudorache D.-I., Bocioagă M., Mihaiescu D.E., Hadibarata T., Grumezescu A.M. (2024). An updated overview of silica aerogel-based nanomaterials. Nanomaterials.

[29] Ivanov K., Filimonova Y.A., Sirotkin N., Agafonov A., Nazarov S. (2024). Hemocompatibility and Antioxidant Properties of Nano-Sized Barium Titanate in Cubic and Tetragonal System. J. Clust. Sci..

[30] Namli S., Guven O., Simsek F.N., Gradišek A., Sumnu G., Yener M.E., Oztop M. (2023). Effects of deacetylation degree of chitosan on the structure of aerogels. Int. J. Biol. Macromol..

[31] Demydov D., Klabunde K.J. (2004). Characterization of mixed metal oxides (SrTiO_3_ and BaTiO_3_) synthesized by a modified aerogel procedure. J. Non-Cryst. Solids.

[32] Shimooka H., Kuwabara M. (1996). Crystallinity and Stoichiometry of Nano-Structured Sol-Gel-Derived BaTiO_3_ Monolithic Gels. J. Am. Ceram..

[33] Xing M.-Y., Yang B.-X., Yu H., Tian B.-Z., Bagwasi S., Zhang J.-L., Gong X.-Q. (2013). Enhanced photocatalysis by Au nanoparticle loading on TiO_2_ single-crystal (001) and (110) facets. J. Phys. Chem. Lett..

[34] Auer S., Frenkel D. (2001). Suppression of crystal nucleation in polydisperse colloids due to increase of the surface free energy. Nature.

[35] Zhao G., Li Z., Cheng B., Zhuang X., Lin T. (2023). Hierarchical porous metal organic framework aerogel for highly efficient CO_2_ adsorption. Sep. Purif. Technol..

[36] Yuan M., Zhu Y., Fu J., Xu S., Wu X., Wang Z., Yuan M., Song Z., Cui S. (2023). Facile synthesis of a novel Zn_2_Ti_3_O_8_ aerogel with porous structure for high-efficient degradation of antibiotics under simulated sunlight. Ceram. Int..

[37] Tan X., Qin J., Li Y., Zeng Y., Zheng G., Feng F., Li H. (2020). Self-supporting hierarchical PdCu aerogels for enhanced catalytic reduction of 4-nitrophenol. J. Hazard. Mater..

[38] Song W., Salvador P.A., Rohrer G.S. (2018). The effect of pH on the photochemical reactivity of BaTiO_3_. Surf. Sci..

[39] Kert M., Skoko J. (2023). Formation of pH-Responsive Cotton by the Adsorption of Methyl Orange Dye. Polymers..

[40] Liu S.X., Qu Z.P., Han X.W., Sun C.L. (2004). A mechanism for enhanced photocatalytic activity of silver-loaded titanium dioxide. Catal. Today..

[41] Li Y., Lu G., Li S. (2002). Photocatalytic transformation of rhodamine B and its effect on hydrogen evolution over Pt/TiO_2_ in the presence of electron donors. J. Photoch. Photobio. A.

[42] Butler M.A., Ginley D.S. (1978). Prediction of flatband potentialsat semiconductor-electrolyte interfaces from atomic electronegativities. J. Electrochem. Soc..

[43] Xian T., Yang H., Di L.J., Dai J.F. (2015). Enhanced photocatalytic activity of BaTiO_3_@ g-C_3_N_4_ for the degradation of methyl orange under simulated sunlight irradiation. J. Alloy Compd..

[44] Andersen T., Haugen H.K., Hotop H. (1999). Binding energies in atomic negative ions: III. J. Phys. Chem. Ref. Data.

[45] Zheng H., Li X., Zhu K., Liang P., Wu M., Rao Y., Jian R., Shi F., Wang J., Yan K. (2022). Semiconducting BaTiO_3_@C core-shell structure for improving piezo-photocatalytic performance. Nano Energy.

[46] Devi L.G., Nithya P.M. (2018). Preparation, characterization and photocatalytic activity of BaTiF_6_ and BaTiO_3_: A comparative study. J. Environ. Chem. Eng..

[47] Xiong X., Tian R., Lin X., Chu D., Li S. (2015). Formation and photocatalytic activity of BaTiO_3_ nanocubes via hydrothermal process. J. Nanomater..

[48] Zhou X.T., Ji H.B., Huang X.J. (2012). Photocatalytic degradation of methyl orange over metalloporphyrins supported on TiO_2_ degussa P25. Molecules.

[49] Wei K., Wang B., Hu J., Chen F., Hao Q., He G., Wang Y., Li W., Liu J., He Q. (2019). Photocatalytic properties of a new Z-scheme system BaTiO_3_/In_2_S_3_ with a core–shell structure. Rsc. Adv..

[50] Jiang S., Zhao R., Ren Z., Chen X., Tian H., Wie X., Li X., Shen G., Han G. (2016). A Reduced Graphene Oxide (rGO)-Ferroelectrics Hybrid Nanocomposite as High Efficient Visible-Light-Driven Photocatalyst. ChemistrySelect.

[51] Mahrsi M.I., Chouchene B., Gries T., Carré V., Girot E., Medjahdi G., Ayari F., Balan L., Schneider R. (2023). Novel ZnO/Ag nanohybrids prepared from Ag+-doped layered zinc hydroxides as highly active photocatalysts for the degradation of dyes and Ciprofloxacin. Colloid. Surf. A.

